# Messy eaters and scavenger hunts: the community ecology of scavenging interactions in a rockpool ecosystem

**DOI:** 10.1098/rsos.261342

**Published:** 2026-10-28

**Authors:** Lola I. L. Weiss, Charlotte S. C. Davis, Maddie Doran, Rebecca Gourlay, Leonie Kay, Allison K. Puckett, Melissa E. Ramsay, Margot Rochelet, Michael M. Webster

**Affiliations:** ^1^ School of Biology, University of St Andrews, St Andrews, UK; ^2^ L'Institut Agro Rennes-Angers, Rennes, France

**Keywords:** competition, landscape of fear, public information, scrounging, social foraging

## Abstract

Carcasses are valuable resources to those scavengers able to access them. While some generalist foragers are poorly equipped to process carcasses themselves, they may exploit the actions of others by feeding on tissues that the latter expose or detach. Carcasses also attract opportunistic predators, which can target other smaller scavengers, as well as feeding on the carcass itself. We studied these interactions using field-based experiments in a rockpool ecosystem. First, we showed that greater numbers of small fishes and mysids were attracted to areas where scavengers, primarily hermit crabs (*Pagurus bernhardus*), were feeding on mussel carcass baits compared to treatments where inaccessible carcasses or no-carcass controls were presented. We termed these secondary scavengers, since we did not see them feeding on the carcass directly. Second, we saw predators attack scavengers more frequently in trials where carcasses were present than where they were absent. They also approached stimulus chambers containing aggregations of hermit crabs more often than empty controls. Nonetheless, predator interactions were, overall, infrequent. For these smaller scavengers, carcass-foraging may be a trade-off between access to a sizeable food resource and competition, and sometimes heightened predation risk, from larger scavengers.

## 1. Introduction

Scavenging of animal carcasses is a key ecological process. The bodies of dead animals support communities of facultative predators and omnivores, specialist carrion-eaters and microbial decomposers [1–5]. Succession defines the consumption and breakdown of carcasses, and the actions of initial scavengers facilitate exploitation by other species. This is true for carcasses that persist for years, such as deep-ocean whale falls [6–8], but also for short-lived carcasses, those that are consumed over timescales of months [9] or even hours [10].

For many generalist scavengers, intact carcasses might not be readily accessible. Scavengers may not be able to break through the integument that surrounds the edible tissues, or they may not be capable of removing smaller, consumable pieces of tissue from larger masses. If they are to feed from the carcass, they may need to wait for other scavenging species that are better equipped to begin the process of breaking apart the body. Many large predators are facultative scavengers, eating carcasses when they encounter them [11–13]. The same adaptations, specialized claws, teeth or beaks, and dextrous and strong limbs and jaws, that allow them to feed on animals that they have predated directly, also allow them to break apart carcasses. Large or robust generalist foragers, too, may be able to tackle intact carcasses in this way. Once these initial scavengers have begun to exploit the carcass, a succession of other species can follow them [9,14].

Selva *et al.* [9] monitored the consumption of carcasses of European bison (*Bison bonasus*) by a mammalian and avian scavenging guild in the Bialowieza Forest in Poland. Here, wolves (*Canis lupus*) played an important role in facilitating access to edible parts of the carcasses by other scavenger species. Most species were not able to open the thick hide of bison to access internal tissues and organs, especially when the carcasses were frozen. Only after wolves had removed parts of the hide were other species able to feed on the underlying musculature. Moreover, the feeding action of the wolves facilitated progressive scavenging by others, since the wolves were also powerful enough to break and move the ribs of the bison, once exposed, allowing other scavengers to access the internal organs within the ribcage.

Davenport *et al.* [10] studied the interactions of scavengers on carcasses of small fish (mackerel, *Scomber scomber*, < 30 cm body length) in a coastal marine environment. They noted a succession of scavenger arrivals, with the most mobile benthic fishes and swimming crabs appearing at the carcasses within minutes, followed later by crawling crabs, and, after tens of minutes to hours, molluscs (whelks, *Nassarius* sp.), starfishes, small sharks and spider crabs. While swimming and crawling crabs, small sharks, and some demersal fishes such as shannies (*Lipophrys pholis*) fed directly on the carcass, others such as sand gobies (*Pomatoschistus minutus*) and whelks gathered in the vicinity of the carcass and fed on smaller pieces of tissue that were detached from the carcass by those feeding on it directly. In particular, Davenport *et al.* [10] noted that crab species generated a lot of detached material that supported indirect scavenging by other species.

Carcasses may also become sites of predation. Many predatory species will also opportunistically forage on carcasses, and carcass sites may bring predators and small scavengers—potential prey—together. Auster *et al.* [15] used remotely operated vehicle-monitoring to record small sharks scavenging on the carcass of a large billfish in the western Atlantic. Wreckfish (*Polyprion americanus*), a larger predatory teleost, was also attracted to the carcass, and one was recorded capturing and consuming a small shark. Moleón *et al.* [16] studied the dynamics of African scavenger assemblages at carcasses of different sizes. They noted that while the overall number of scavenger species increased with carcass size, the number of small mammalian scavengers remained stable. They attributed this pattern to the perception of larger carcasses as sites associated with higher predation risk by mesocarnivores, since large predators visited these frequently. Carcasses may function both as a resource and as a predation risk hotspot for smaller scavengers.

In this study, we investigated two aspects of carcass scavenging: facilitation of feeding by generalist scavengers (part 1) and scavenger interactions with predators (part 2). We conducted these experiments using baited cameras in a rocky shore ecosystem where European hermit crabs (*Pagurus bernhardus*) are the most common scavenger species. European hermit crabs are generalists that exploit a broad range of resources, sifting sand for detritus [17], filtering suspended plankton [18], feeding on larger invertebrate prey [19] and consuming the carcasses of molluscs and fishes [20]. In a previous investigation, we saw that foraging by hermit crabs on mollusc carcasses generated particle plumes that acted as a form of inadvertent social information, attracting further hermit crabs to the location of the carcass [21]. Potentially, other species may also be attracted to these feeding aggregations by plumes of detached tissue fragments.

In part 1, we studied this directly, using camera rigs that were either baited with accessible carcasses (de-shelled mussels, *Mytilus edulis*), inaccessible carcasses (placed within cage-like containers so that they could be detected by scavengers, but not physically reached), or no-bait controls. We predicted that not only would more hermit crabs be attracted in the accessible carcass treatment than in the inaccessible carcass and no-carcass treatments, but also that greater numbers of secondary scavenging species of small fishes and pelagic crustaceans would be attracted in the accessible carcass treatment than in the other two treatments too. We made these predictions reasoning that the feeding actions of the primary-scavenging hermit crabs would produce social cues in the form of particle plumes, attracting further primary and secondary scavengers as in Rowe *et al.* [21].

In part 2, we focussed on scavenger-predator interactions. We presented (a) baited and unbaited camera rigs, and (b) stimulus chambers containing simulated hermit crab aggregations in the absence of a carcass bait, as well as empty stimulus chambers as a control. We predicted that we would see greater numbers of both hermit crabs and larger scavengers—potential predators—around the baited rigs than at the unbaited rigs. We also predicted that attacks on hermit crabs by predators would be more frequent around the baited rigs. Finally, we predicted that potential predators would be drawn to simulated hermit crab aggregations more often than to empty stimulus chambers.

## 2. Methods

### 2.1. Study site

These experiments were carried out in several rockpools on the rocky shore of East Sands beach in St Andrews, UK. Trials were performed in daylight and typically within 2 hours either side of low tide (or within 1 hour for the lower shore). Part 1 took place between December 2023 and March 2024 (hereafter winter) and May 2024 and July 2024 (hereafter spring/summer). Part 2 took place between June and September 2025 (all of experiment (a) and the summer trials of experiment (b)) and January and April 2026 (the winter trials of experiment (b)). We used a total of six rockpools in part 1. These sat in discrete depressions on the shore, but were sometimes connected, depending upon the height of the tide, and were inundated completely twice daily. Because of this, we classified the rockpools as lower-shore (two pools) or mid-shore (four pools) in our analyses below, but did not consider them separately. In part 2, we only used the lower-shore rockpools, where predators were more common. The coordinates of the rockpools are provided in the supplementary material. The surface area of the rockpools varied from an estimated minimum of 30 to 400 m^2^. They were typically 5–60 cm deep (one lower-shore pool had a maximum depth of around 120 cm) and we focussed on areas of 30–90 cm depth when deploying experimental apparatus. The beds of the rockpools were a mixture of sandstone bedrock, coarse sand or pebbles, with larger boulders and patches of macroalgae. When placing apparatus, we selected open areas with a flat bed that allowed us to set the rigs flat and afforded an uninterrupted view for the cameras. Hermit crabs were abundant at the study site (estimated at > 20 m^–2^, [21]). A number of other crustaceans and fish species were also present in the rockpools, with juvenile and adult green shore crabs (*Carcinus maenas*), common prawns (*Palaemon serratus*), mysids (*Leptomysis* spp.), sculpin (*Myoxocephalus scorpius*), two spotted gobies (*Gobiusculus flavescens*), rock gobies (*Gobius paganellus*), juvenile whiting (*Merlangius merlangus*), shannies (*Lipophrys pholis*), flounders (*Platichthys flesus*) and corkwing wrasse (*Symphodus melops*) recorded in a previous study [21]. Of these, larger green shore crabs (carapace width > 4 cm), sculpin, shannies and adult flounders were considered potential predators of hermit crabs and other small scavengers.

### 2.2. Part 1. Messy eaters: primary scavengers facilitate secondary scavenging

#### 2.2.1. Experimental apparatus

Data were collected using an underwater camera rig consisting of a base made from stainless-steel fixing plate arms (55 cm long) arranged in a right angle and held in place by a diagonal cross-brace attached to either arm. A further cross-brace ran from the apex of the two arms to the centre of the first, diagonal cross-brace (figure 1). A stainless-steel cube-shaped frame (27 × 27 × 27 cm^3^) formed a three-dimensional ‘survey volume’, within which we recorded scavengers. This was attached to the rig, so that the apex of the two arms was at its centre. Two GoPro Hero 5 cameras with screw-on housing mounts, attached to a 10 cm bolt, placed at either end of each arm allowed us to record simultaneously and to determine whether attendant scavengers were inside or outside the survey volume. A binder clip and a holding tube were placed centrally within the cube frame. These were used to hold the carcass baits, as described below. The holding tube was made from a swim feeder (a device used by anglers to drop food close to the area being fished) and consisted of a 5.5 cm long and 2.5 cm diameter green plastic tube, containing twenty-one 0.5 cm diameter round holes. An inflatable tow float (oval-shaped, 40 cm long) was attached to each rig via a 1.5 m long nylon rope for ease of retrieval. In some trials, we used a similarly sized balloon as a marker float. Two of these rigs were built and were used simultaneously in the experiments described below.

**Figure 1.**
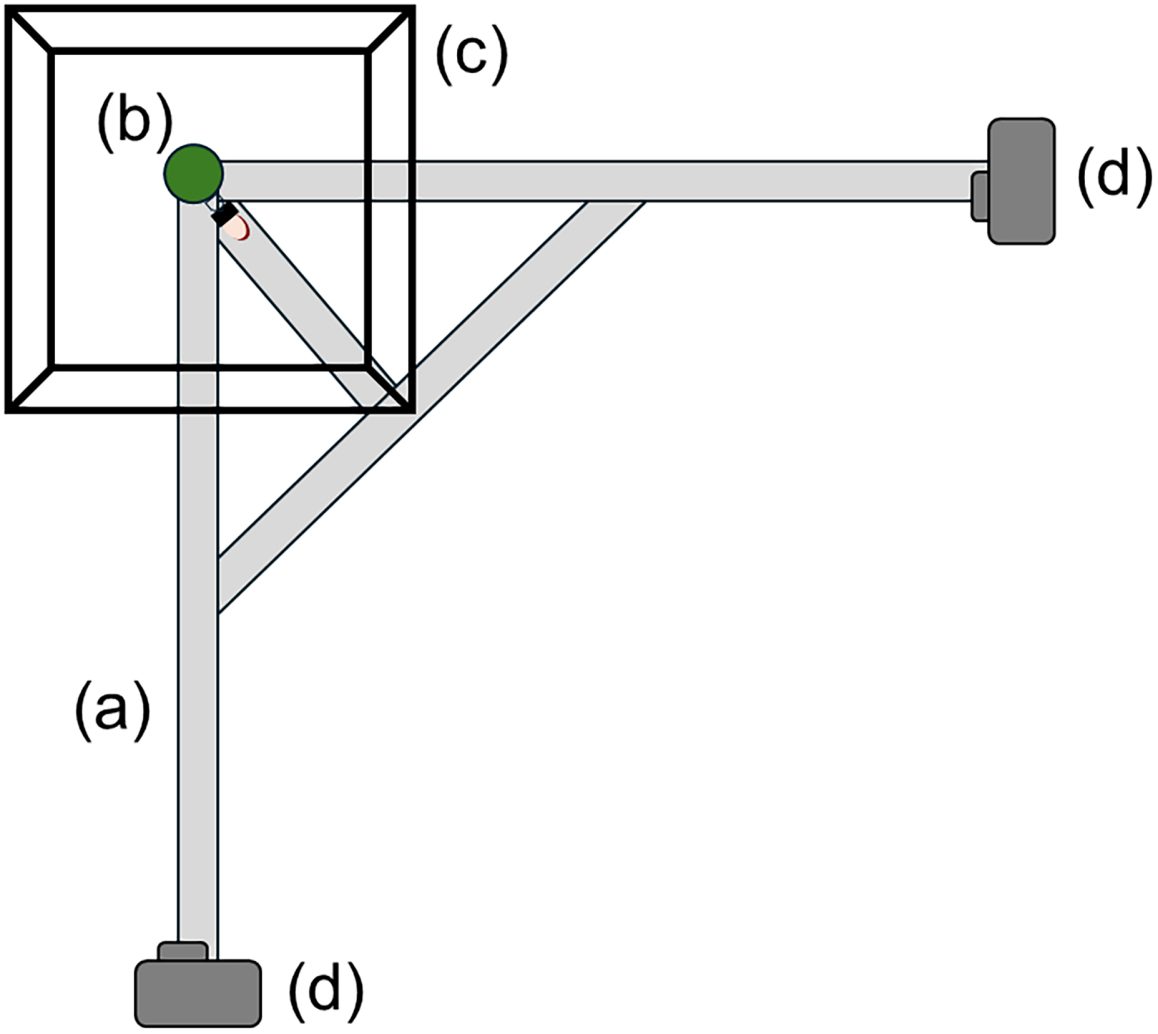
A diagram of the experimental rig used in part 1. The base of the rig (a) consisted of stainless-steel fixing plates. A carcass bait (de-shelled mussel) was held in place at location (b). A holding tube was also placed here, with the mussel either located outside of this (accessible treatment) or within it (inaccessible treatment). In the control, no mussel carcass was presented. A wire-framed cube (c) delineated the three-dimensional survey volume, within which we counted all primary and secondary scavenging species. Two GoPro Hero 5 cameras on either arm of the rig (d) allowed us to record simultaneously and to determine whether attendant scavengers were inside or outside of the survey volume. The diagram is approximately drawn to scale; each arm of the rig was 55 cm long.

#### 2.2.2. Experimental treatments

We performed three treatments: one in which the carcass was placed in the binder clip and could be fed upon by scavengers (‘accessible’), one in which the carcass was placed inside the swim feeder tube and could be detected but not fed upon by scavengers (‘inaccessible’) and a third treatment in which no carcass was present (‘control’). The carcasses were thawed and de-shelled mussels (*Myt. edulis*) were purchased from Tesco supermarket. They weighed 2.79 ± 0.04 g (thawed weight, mean and standard error, *n* = 20). We performed 15 replicates per treatment per season, for a total of 90 trials.

#### 2.2.3. Experimental procedure

The order of the 45 trials within each season was randomly predetermined. Selection of rockpools was more opportunistic, as those on the lower shore were sometimes inundated by the tide. Trials were always performed within 2 hours of low tide, and for each trial, we recorded whether the tide was rising or falling. Typically, we used two rigs. We did not place these in the smaller pools simultaneously, but we did sometimes deploy two rigs at once in the larger pools. When we did so, we ensured that these were placed at least 10 m apart. Each trial ran for 30 minutes. The rigs were walked out to a relatively flat and unobstructed area of the rockpool, 30–60 cm deep. The cameras were switched on (1920 × 1080 resolution, 30 frames per second) and the rig was gently placed on the bed of the rockpool, with the inflatable float positioned downwind, so that it did not drift over the rig. After 30 minutes, we retrieved the rig. From the resulting videos, we disregarded the first 10 minutes to allow disturbed sediment to settle, extracting data from the remaining 20 minutes.

#### 2.2.4. Data processing

For the 20 minute sampling period, we recorded the number of primary scavengers (predominantly hermit crabs, but also smaller green shore crabs, carapace size estimated at 10–20 mm, and common prawns) that were present within the survey volume of the cube, and the number of these that were actively feeding on the carcass (in the accessible treatment only). To do this, we used scan sampling. For 20 seconds every minute, we counted the number of hermit crabs within the cube. This yielded 20 counts per trial.

We also recorded the presence of other fish and crustacean species within the cube. These were recorded continuously, with the species, arrival and departure time recorded for each visit. In some cases, we could see these feeding on pieces of detached mussel, but in many trials it was not possible to see if they were feeding or not. Accordingly, we recorded the presence of all other fish and crustacean species within the survey volume, whether feeding or not.

We recorded a categorical estimate of water clarity. Sometimes suspended fine sediment made the water slightly turbid, which might have affected our estimates of scavenger abundance. Since we had no means of accurately quantifying turbidity in the field, we classified the trial as clear water (apparatus fully visible with no apparent coloration in the water) or murky (some cloudiness apparent, but apparatus still visible). We did not perform trials if the far end of the cube delineating the survey volume was not clearly visible. Finally, we recorded the temperature in the rockpools to the nearest 0.1°C. This was taken at the end of each trial. Temperature data were not obtained for 8 out of 45 trials in the winter season. Temperature in the winter season ranged from 4.9 to 10.9°C. In the spring/summer season, it ranged from 12.5–17.0°C. All analyses described below were conducted using RStudio [22].

#### 2.2.5. Statistical analysis

*Primary scavenger arrivals*. We first compared the number of primary scavengers recorded per minute between each of the three treatments (carcass accessible, inaccessible and no-carcass control) using a general linear model with a Poisson distribution. Hermit crabs, juvenile green shore crabs and common prawns were the only species seen to feed directly upon the carcass in the accessible treatment or to gather close to it in the inaccessible treatment. Hermit crabs were by far the commonest primary scavenger (98% of scavengers). All three species were pooled for analysis and counted as primary scavengers. Treatment was included as a fixed factor, along with season (winter or spring/summer), location on shore (lower or mid), water clarity (clear or turbid) and tide (rising or falling). Temperature was included as a continuous covariate. We also included an interaction between treatment and season. Time (sampling point within trial) was also included. We performed post hoc tests for the effect of treatment using Tukey's HSD post hoc comparisons.

*Secondary scavenger arrivals*. We compared the number of secondary scavengers recorded per minute, pooling all species, between each of the three treatments using a general linear model with a Poisson distribution. Treatment was included as a fixed factor, along with season (winter or spring/summer), location on shore (lower or mid), water clarity (clear or turbid) and tide (rising or falling). Temperature was included as a continuous covariate. We also included an interaction between treatment and season. Time (sampling point within trial) was included, as was the number of primary scavengers present in each minute. We performed post hoc tests for the effect of treatment using Tukey's HSD post hoc comparisons.

### 2.3. Part 2. Scavenger hunt: scavenger-predator interactions at carcasses

#### 2.3.1. Scavenger and predator attraction to baited and unbaited rigs

*Experimental apparatus*. Cameras were mounted to rigs consisting of a strip made from stainless-steel fixing plate arms (65 cm long) with a cross-brace attached 10 cm from one end for stability. A camera (either GoPro Hero 5 or Akaso EK7000) with a screw-on housing mount was attached to the top of a 10 cm bolt, placed at one end of the rig. A binder clip was attached to the opposite end using a plastic cable tie (figure 2). The binder clip was used to hold the carcass bait, as described below. An inflatable tow float (40 cm long) was attached to each rig via a 2 m long nylon rope for ease of retrieval. Up to three rigs were used simultaneously in the experiments described below.

**Figure 2.**
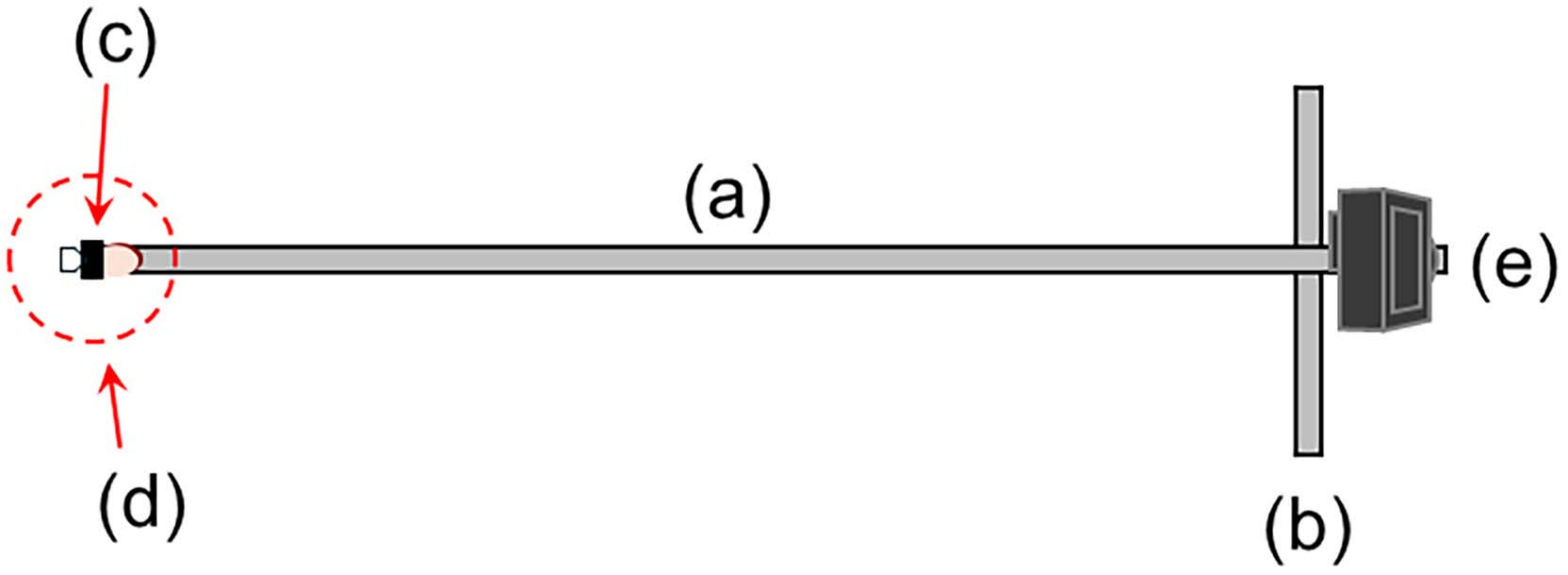
Plan-view diagrams of the apparatus used in the first experiment of part 2. (a) A 65 cm long stainless-steel fixing plate. (b) A 20 cm fixing plate attached at 90 degrees for stability. (c) A binder clip attached to the fixing plate using a plastic cable tie. In the baited treatment, it held a de-shelled mussel, while in the unbaited treatment it was empty. Panel (d) indicates a 10 cm diameter zone within which we counted hermit crabs and interactions between these and potential predators. (e) GoPro Hero 5 or Akaso EK7000 camera with screw-on mount. See main text for further details.

*Experimental treatments and procedure*. We performed two treatments: one in which the carcass was placed in the binder clip and could be fed upon by scavengers (‘baited’) and a second treatment in which no carcass was present (‘unbaited’). The carcasses were thawed and de-shelled mussels as described for part 1. We performed 27 replicates for the baited treatment and 31 for the unbaited treatment. Treatment type was randomly assigned. We only performed experiments on the lower shore, where rockpools tended to be larger and deeper, and we had previously observed more predators.

Trials were always performed within 1 hour of low tide, when the lower shore was accessible. When placing rigs, we ensured that these were placed at least 10 m apart. We allowed trials to run for as long as possible, with trial lengths limited by camera battery life and the rising tide. Trial lengths ranged from 26 to 55 minutes, with a median duration of 44 minutes. The rigs were walked out to a relatively flat and unobstructed area of the rockpool, 30–90 cm deep, with sand, gravel or bedrock substrate. The cameras were switched on (1920 × 1080 resolution, 30 frames per second) and the rig was gently placed on the bed of the rockpool, with the inflatable float positioned downwind, so that it did not drift over the rig. At the end of each trial, we recorded the temperature in the rockpools to the nearest 1°C. Temperature ranged from 11–15°C.

*Data processing*. We extracted data from the full duration of each video. For each video, we recorded the number of hermit crabs that were present within 5 cm of the clip binder (which held the mussel carcass in the baited treatment and was empty in the unbaited treatment). We counted the number of hermit crabs in the zone around the clip binder every minute. From these data, we calculated the mean number of hermit crab visits per minute per trial. Other small scavengers occurred in lower numbers, including small green shore crabs, carapace size estimated at 10–20 mm, and common prawns, but these were not included in our analyses. Species identified as secondary scavengers in part 1 (juvenile whiting, two spot gobies and Mysid species) were also seen, but again these were not counted.

We performed a full count of predatory species in each video. We included predator approaches, where the predator was seen within approximately 50 cm of the clip binder. We also recorded predator behaviour, noting whether they fed on the carcass (in the baited trials only), and whether they attacked hermit crabs within 5 cm of the clip binder. We saw green shore crabs and shannies attack hermit crabs. For green shore crabs, an attack was considered to have occurred if they seized and handled a hermit crab in their claws, regardless of whether they predated or discarded it. For shannies, an attack consisted of a burst of acceleration towards the hermit crab, resulting in contact. Finally, as the water clarity was judged to be high in all trials, we did not include a categorical estimate of water clarity, as we did in part 1.

*Statistical analysis*. We used three general linear models to investigate scavenger abundance, predator approaches and predator attacks. We first compared the mean number of hermit crabs per minute that were present within 5 cm of the clip binder, fitting a model with a Poisson distribution. We included treatment as a fixed factor and water temperature as a continuous covariate. Next, we compared the number of predator approaches between the two treatments, this time fitting a model with a zero-inflated Poisson distribution and again including water temperature as a continuous covariate. Because the majority of predators seen were green shore crabs, we did not include predator species in the model. This information is presented qualitatively in the results. Finally, we compared the number of predator attacks on hermit crabs, again fitting a zero-inflated Poisson distribution, and including treatment as a fixed factor and water temperature as a continuous covariate.

#### 2.3.2 Predator attraction to hermit crab aggregations in absence of carcasses

*Experimental apparatus*. We used a camera rig consisting of a stimulus chamber attached to one end of the stainless-steel fixing plate, and a camera at the other. The stimulus chamber held aggregations of five, 10 or zero (control) hermit crabs (figure 3). The stimulus chamber consisted of a Perspex box measuring 15.0 × 6.2 × 7.6 cm^3^. We drilled three 10 mm holes in the front of the chamber, 20 mm apart. We also drilled five 10 mm holes in the base. Four of these were 20 mm in from each corner. The fifth was in the centre and was used to bolt the chamber to a fixing plate, described below. The lid had two 10 mm holes, 30 mm apart from each other in the centre. The holes facilitated water flow between the chamber and surrounding water, allowing chemical cues to leave the chamber. The chamber was attached to a stainless-steel fixing plate measuring 65 cm long and 2.5 cm wide using stainless-steel wingnuts and bolts. A second T-shaped fixing plate measuring 20 cm wide by 10 cm long was bolted to the first to provide stability. At the opposite end of the apparatus from the stimulus chamber was a 50 mm bolt, placed thread-end up. We used this to screw on a camera (GoPro Hero 5 or Akaso EK7000 with screw-on housing mount). We constructed three of these devices and used either two or three simultaneously when performing trials. We attached an inflatable tow float (40 cm long) as a buoy to each apparatus using a 1.5 m nylon rope attached to the end of the rig where the camera was located. This was extended away from the rig in a downwind direction to prevent it from drifting over the apparatus.

**Figure 3.**
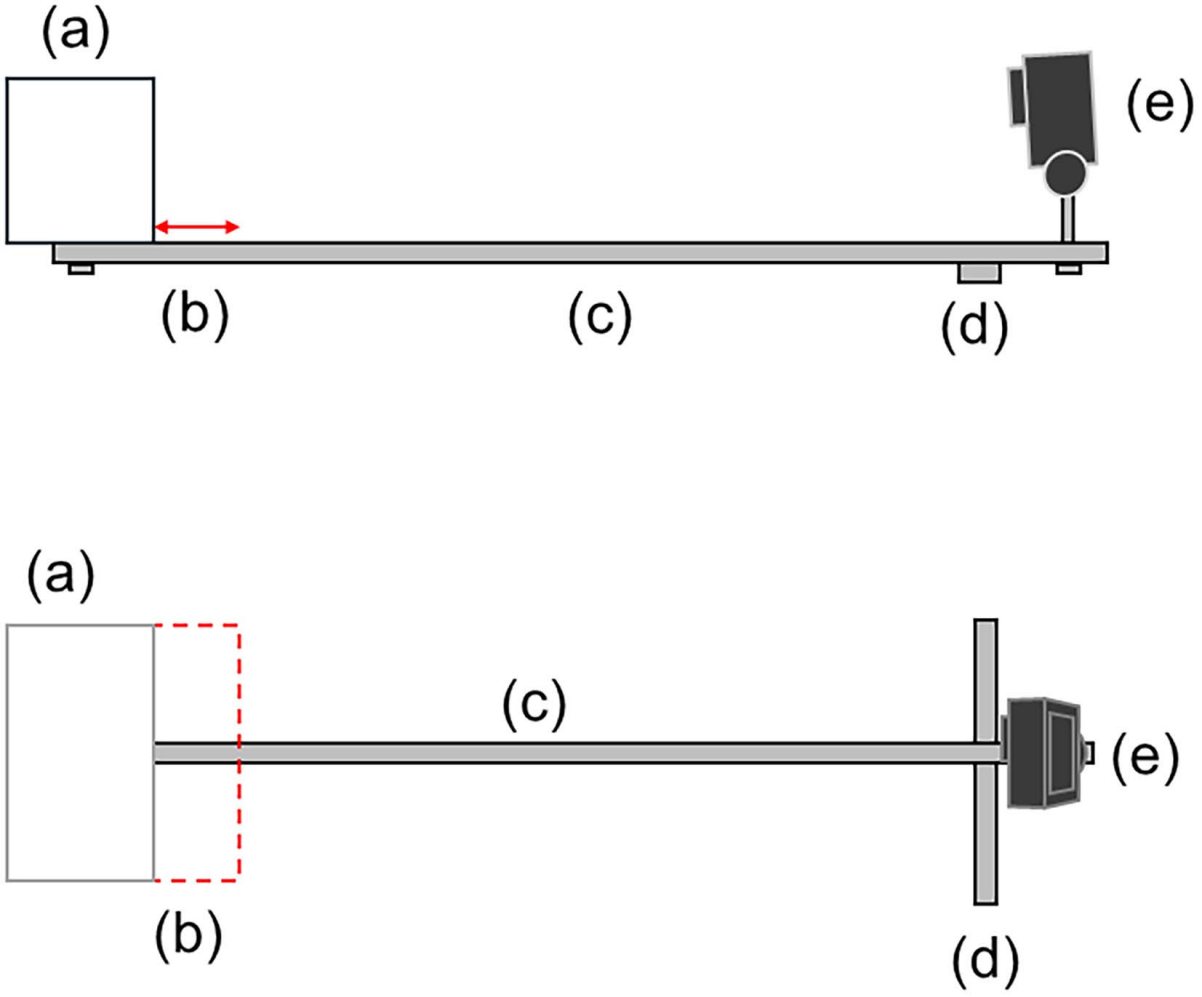
Profile and plan-view diagrams of the apparatus used in the second experiment of part 2, based on Rowe *et al.* [21]. (a) The stimulus chamber (15.0 × 6.2 × 7.6 cm^3^) used to hold aggregations of zero (control), five or 10 hermit crabs. (b) The stimulus goal zone within which attracted hermit crabs were counted (note that this was not physically marked but was delineated using a drill hole in the fixing plate). (c) A 65 cm long stainless-steel fixing plate. (d) A 20 cm fixing plate attached to the 65 cm fixing plate at 90 degrees, for stability. (e) GoPro Hero 5 or Akaso EK7000 camera with screw-on mount. Predator attacks directed to any side of the stimulus chamber were recorded. See main text for further details.

The hermit crabs placed in the stimulus chambers were collected from the margins of the rockpools, held in a bucket of seawater for no more than 10 minutes and were then added to the stimulus chambers immediately before they were deployed. We collected hermit crabs occupying common periwinkle (*Littorina littorea*) shells that were between 15 and 20 mm tall. More than 90% of the hermit crabs at this location were found in common periwinkle shells, most within this size range. The stimulus hermit crabs were released at the point of collection after each trial.

*Experimental treatments and procedure*. We investigated whether predators were attracted to simulated aggregations of hermit crabs in the absence of carcass baits. We compared attraction rates to empty stimulus chambers and chambers containing five or 10 hermit crabs. In total, we performed 25 replicates per treatment, running trials between June and September 2025 (‘summer’) and January and March 2026 (‘winter’). For the zero, five and 10 hermit crab aggregation treatments in summer 2025, there were, respectively, 11, 11 and 12 replicates, and for winter 2026 there were 14, 14 and 13.

Treatment order was randomly predetermined. We only performed experiments on the lower shore where rockpools tended to be larger and deeper, within 1 hour of low tide. Rigs were placed at least 5 m apart. We allowed trials to run for as long as possible, determined by camera battery life and the rising tide. Trial lengths ranged from 21 to 56 minutes, with a median duration of 39 minutes. The rigs were set in flat and unobstructed areas of the rockpools, 30–90 cm deep, with sand, gravel or bedrock substrate. The cameras recorded at 1920 × 1080 resolution and 30 frames per second. At the end of each trial, we recorded the temperature in the rockpools to the nearest 1°C. Temperature ranged from 6 to 15°C.

*Data processing*. We extracted data from the full duration of each video. We first recorded the number of hermit crabs that were attracted to the area containing the simulated aggregation. We did this for two reasons: first because they might use the aggregation as a social cue of resource presence (though Rowe *et al*. [21] found no such effect), and second because additional hermit crabs outside the stimulus chamber effectively increase the overall size of the aggregation and need to be accounted for in models of predator attraction. (This ultimately proved unnecessary, as hermit crabs were not attracted in sizeable numbers in any treatment.) To do this, we designated a recording zone in front of the stimulus chamber, using a drilled hole in the fixing plate to demarcate an area extending to 5 cm in front of the stimulus chamber as a goal zone (figure 3). We counted the number of hermit crabs every minute in the recording zone. From these data, we calculated the mean number of hermit crab visits per minute per trial.

Next, we recorded approaches by predatory species, noting when predators were seen within approximately 50 cm of the stimulus chamber. We also recorded predator ‘contacts’ on the stimulus chambers, using these as a proxy for attacks. These were all performed by adult green shore crabs and took the form of crabs grappling and attempting to manipulate the stimulus chamber with their claws. As in experiment (a), the water clarity was judged to be high in all trials, so we did not include a categorical estimate of water clarity.

*Statistical analysis*. We created three general linear models to compare hermit crab attraction to the aggregations, predator approaches and predator contacts. We first compared the mean number of hermit crabs per minute that entered the recording zone in front of each chamber, using a model with a Poisson distribution. Treatment (zero, five or 10 hermit crabs inside the stimulus chamber) was included as a fixed factor, along with season (summer or winter). Temperature was included as a continuous covariate. We also included an interaction between treatment and season. Second, we compared predator approaches between treatments. Here, we used a model with a zero-inflated Poisson distribution to account for the fact that no predator approaches were seen in many trials. We included treatment and season as fixed factors and temperature as a continuous covariate. We again included an interaction between treatment and season and performed Tukey's HSD post hoc tests for effects of treatment. Third, we compared predator contacts with the stimulus chamber, again using a model with a zero-inflated Poisson distribution, including treatment and season as fixed factors and temperature as a continuous covariate.

*Limitations statement*. The STRANGE reporting framework encourages researchers to declare and discuss potential sources of bias that might limit test subject representativeness and generalizability from their study sample to the wider population or other species [23,24]. In this study, primary and secondary scavenging species were free to approach or avoid the experimental apparatus, which was placed in open areas, so as to have uninterrupted lines of sight from the cameras to the area surrounding the stimulus. In doing so, we may have included bolder or more motivated scavengers in our sample, since earlier work found that hermit crabs collected from open areas behaved differently than those collected from beneath cover [25]. We also used a short settling period and short observation period of 10 and 20 minutes, respectively, in part 1 and no settling period in part 2, which may have reduced visits by shy individuals. We have no cause to believe that this may have affected the differences in scavenger recruitment that we saw between treatments, but this potential limitation should be noted.

While the primary and secondary scavenging species were all abundant at the location of our study, we cannot exclude the possibility that the same individuals were recorded more than once. It was not feasible to mark animals at the study site, and none had any obvious features that could be used for individual recognition. We have no data on either site fidelity or dispersal in any of these species that might provide insight into potential for repeated visits. We are confident that our experimental design, which spread the experiment over several months and used rigs placed in different areas of the lower-shore and mid-shore, should have reduced the potential for pseudoreplication, but we cannot exclude this possibility completely.

While we report an effect of season on secondary scavenger visit rates and predator interaction rates, below, we highlight that we do not have data spanning multiple years and that we only focussed on a single location, so this finding should be interpreted accordingly. We also did not account for lunar phase in our experiments. Lunar rhythms affect a diverse range of behaviours in coastal animals, through both responses to tide state and via endogenous biological clocks [26].

For the experiments described here, it was not possible to record data blind. Data were extracted manually from videos in which the experimental apparatus, and therefore treatment type, were visible at all times.

## 3. Results

### 3.1. Part 1. Messy eaters: primary scavengers facilitate secondary scavenging

#### 3.1.1. Primary scavenger arrivals

We found a significant difference in primary scavenger numbers between treatments (table 1; figure 4), specifically that they were present more in the accessible carcass treatment than in the inaccessible treatment (Tukey's HSD post hoc, *p* = 0.001) or no-carcass control (*p* < 0.001), while more were present in the inaccessible than the control treatment (*p* = 0.006). There was no difference between winter and summer trials, no effect of location on the shore, time of trial relative to rising or falling tide, nor any effect of water clarity or temperature. There was no effect of trial time, with no increase or decrease in primary scavenger numbers apparent over the course of the trial.

**Table 1.** General linear model of factors affecting primary scavenger abundance.

	d.f.	*F*	*p*	$\eta_{p}^{2}$
intercept	1	151.93	<0.001	
season	1	0.71	0.40	0.01
treatment	2	22.95	<0.001	0.39
season*treatment	2	0.93	0.34	0.03
time	19	0.65	0.87	0.01
upper/lower shore	1	2.44	0.19	0.03
water clarity	1	0.28	0.59	0.01
tide rising/falling	1	2.16	0.15	0.03
water temperature	1	0.25	0.47	0.01

**Figure 4.**
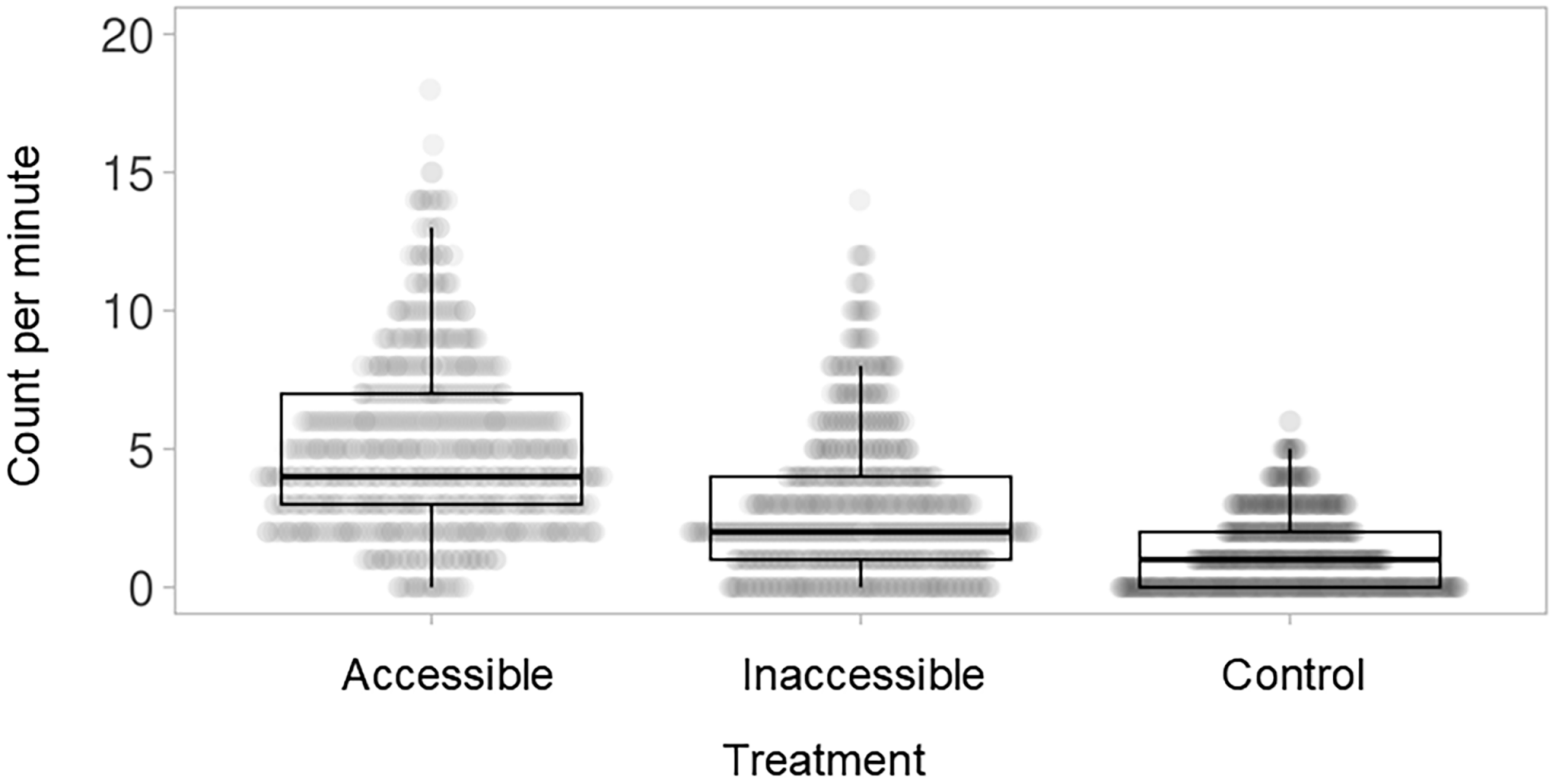
More primary scavengers, predominantly hermit crabs, were present per minute when the mussel carcass was accessible than when it was inaccessible or in the control condition, where no carcass was present. Box plots depict median, interquartile range and 95% confidence intervals. Points depict individual counts.

#### 3.1.2. Secondary scavenger arrivals

Secondary scavenger species recorded were juvenile whiting, two-spot gobies, fifteen-spine sticklebacks and unidentified mysids of *Leptomysis* spp. Numbers varied between species and between seasons. While two-spot gobies were common in both winter and spring/summer (164 and 106, respectively), mysids were more common in spring/summer (139) than winter (10), and juvenile whiting were only seen in the spring/summer (905), where they were highly abundant. Only two fifteen-spine sticklebacks were seen, both in the summer.

We saw a substantial effect of treatment on secondary scavenger numbers (table 2; figure 5), with more seen in the carcass accessible treatment than in the carcass-inaccessible (Tukey's HSD post hoc, *p* < 0.001) or no-carcass (*p* = 0.002) treatments, with no difference between the latter two (*p* = 0.96). There was also an interaction between season and treatment, with more secondary scavengers recorded in the accessible mussel treatment in the spring/summer than in the winter. There was no tendency for more secondary scavengers to arrive over time as the trial progressed. There was no relationship between the average number of primary scavengers present and secondary scavenger numbers. There was also no effect of location on the shore, time of trial relative to rising or falling tide, water clarity or temperature on secondary scavenger numbers.

**Table 2.** General linear model of factors affecting secondary scavenger abundance.

	d.f.	*F*	*p*	$\eta_{p}^{2}$
intercept	1	151.93	<0.001	
season	1	3.63	0.061	0.01
treatment	2	6.65	0.002	0.04
season*treatment	2	5.25	0.007	0.03
time	19	0.37	0.994	0.01
primary scavengers present	1	0.35	0.554	0.01
upper/lower shore	1	0.12	0.717	0.01
water clarity	1	3.03	0.089	0.01
tide rising/falling	1	0.66	0.889	0.01
water temperature	1	0.19	0.740	0.01

**Figure 5.**
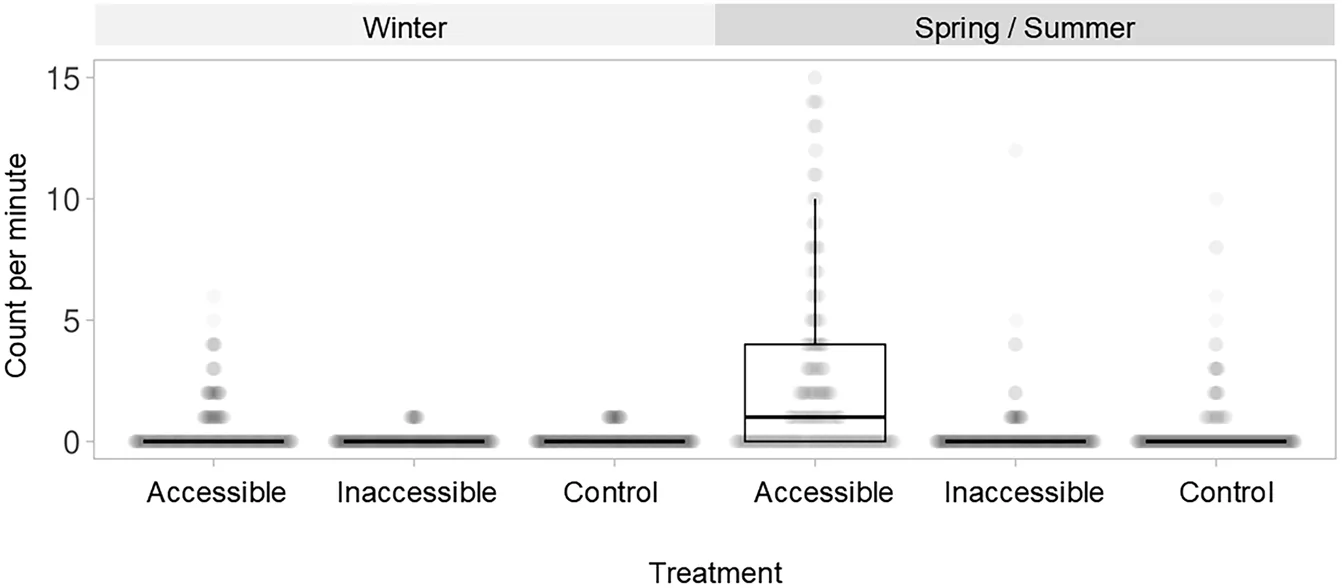
More fish and invertebrate secondary scavengers, predominantly juvenile whiting, two-spot gobies and mysids, visited when the mussel carcass was accessible than when it was inaccessible or in the control condition, where no carcass was present. There was an interaction between treatment and season, with more secondary scavengers seen in the accessible carcass treatment in the spring/summer trials than during winter. Box plots depict median, interquartile range and 95% confidence intervals. Points depict individual counts.

### 3.2. Part 2. Scavenger hunt: scavenger-predator interactions at carcasses

#### 3.2.1. Scavenger and predator attraction to baited and unbaited rigs

We obtained 27 and 31 videos of baited and unbaited rig deployments, totalling 1123- and 1304-minute duration, with a median trial duration of 44 and 45 minutes, respectively. As expected, more hermit crabs were seen in the zone around the clip binder in the baited trials compared to unbaited (general linear model (GLM), *F*_1,55_ = 61.91, *p* < 0.001, $\eta_{p}^{2}$ = 0.52; figure 6a). We saw no effect of water temperature on hermit crab visit numbers (*F*_1,55_ = 1.55, *p* = 0.28, $\eta_{p}^{2}$ = 0.01).

**Figure 6.**
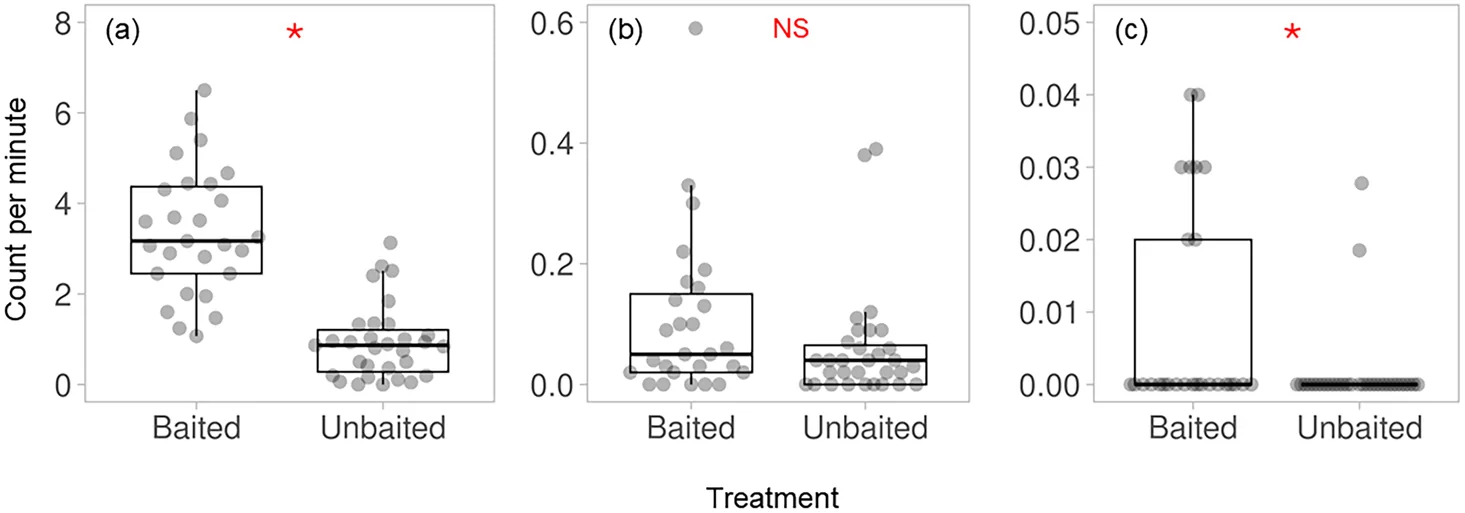
(a) Hermit crab visits, (b) predator approaches, and (c) predator attacks on hermit crabs per minute, at baited and unbaited rigs. Asterisk (*) denotes significant effect of treatment, NS indicates no effect. Box plots depict median, interquartile range and 95% confidence intervals. Points depict individual counts.

Focussing on predator visits, we recorded 119 and 75 predator approaches in the baited and unbaited treatments. We pooled predator species. Green shore crabs were the commonest predator species, accounting for 82 and 75% of sightings in the baited and unbaited treatments, respectively (table 3). We saw no difference in predator approach rates between treatments (figure 6b), nor any effect of water temperature (GLM, treatment: *F*_1,55_ = 2.24, *p* = 0.13, $\eta_{p}^{2}$ = 0.04, temperature *F*_1,55_ = 0.01, *p* = 0.97, $\eta_{p}^{2}$ = 0.01).

**Table 3.** Counts of different predator species seen to approach the baited and unbaited rigs in experiment 2(a) and at the simulated aggregations of zero, five or 10 hermit crabs in experiment 2(b).

	predator species: raw counts (counts per minute)				
	green shore crab	shanny	sculpin	flounder	rock goby
(a) scavenger and predator attraction to baited and unbaited rigs					
baited	98 (0.087)	11 (0.010)	5 (0.004)	5 (0.004)	0 (0)
unbaited	56 (0.043)	8 (0.006)	3 (0.002)	7 (0.005)	1 (0.001)
(b) predator attraction to hermit crab aggregations in absence of carcasses					
zero	1 (0.001)	0 (0)	0 (0)	0 (0)	0 (0)
five	36 (0.036)	2 (0.002)	2 (0.002)	2 (0.002)	0 (0)
10	21 (0.022)	2 (0.002)	1 (0.001)	1 (0.001)	1 (0.001)

While predator attacks on hermit crabs were rare, we nevertheless saw significantly more attacks at baited rigs compared to unbaited rigs. In total, we saw 10 attacks by predators (nine by green shore crabs and one by a shanny) in the baited treatment, compared to two attacks (both by green shore crabs) in the unbaited treatment (GLM: treatment: *F*_1,55_ = 5.504, *p* = 0.022, $\eta_{p}^{2}$ = 0.10, temperature *F*_1,55_ = 0.60, *p* = 0.44, *$\eta_{p}^{2}$* = 0.01; figure 6c). By contrast, in the baited treatment we saw 60 instances of the predators (56 by shore crabs and two each by shannies and sculpins) feeding on the mussel.

#### 3.2.2. Predator attraction to hermit crab aggregations in absence of carcasses

We performed 25 trials for each treatment (zero, five or 10 hermit crabs in the stimulus chamber). Trial length ranged from 21 to 56 minutes, with a median length across the three treatments of 39 minutes. First, we compared the number of hermit crabs that were recorded in front of the stimulus chambers, recording no differences between treatments (GLM, *F*_2,68_ = 2.14, *p* = 0.127, $\eta_{p}^{2}$ = 0.05; figure 7a). We also saw no effect of season (*F*_1,68_ = 0.03, *p* = 0.858, $\eta_{p}^{2}$ = 0.001), no interaction between treatment and season (*F*_2,68_ = 2.26, *p* = 0.122, $\eta_{p}^{2}$ = 0.06), nor any effect of water temperature (*F*_1,68_ = 0.05, *p* = 0.821, $\eta_{p}^{2}$ = 0.001) on hermit crab counts.

**Figure 7.**
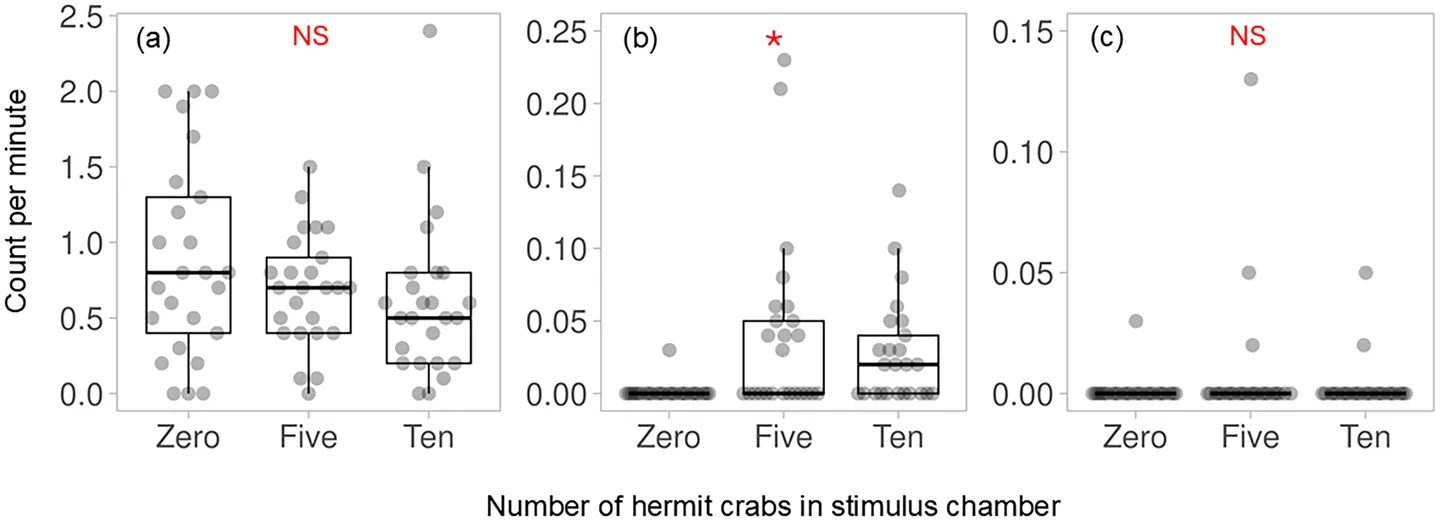
(a) Hermit crab visits, (b) predator approaches, and (c) predator contacts with stimulus chamber per minute, at stimulus chambers containing simulated aggregations of zero, five or 10 hermit crabs. Asterisk (*) denotes a significant effect of treatment, and NS indicates no effect. Box plots depict median, interquartile range and 95% confidence intervals. Points depict individual counts.

Next, we focussed on predator interactions. Again, green shore crabs were the commonest predator, accounting for 84% of sightings across the three treatments (table 3). We saw more predator approaches in the five-hermit crab treatments compared to the zero-hermit crab treatment (GLM, *F*_2,68_ = 9.90, *p* < 0.001, $\eta_{p}^{2}$ = 0.16, Tukey's HSD post hoc tests: zero compared to five *p* < 0.001, zero compared to 10 *p* = 0.050, five compared to 10 *p* = 0.153; figure 7b). We also saw an effect of season, with more approaches in the summer than in winter/spring (*F*_1,68_ = 7.41, *p* = 0.008, $\eta_{p}^{2}$ = 0.07), and an interaction between treatment and season (*F*_2,68_ = 7.14, *p* = 0.002, $\eta_{p}^{2}$ = 0.12). There was no effect of water temperature (*F*_1,68_ = 2.94, *p* = 0.092, $\eta_{p}^{2}$ = 0.02).

Finally, we compared predator contacts with the stimulus chamber. These were rare, with only six seen in total across the 45 trials. Here, we saw no difference in predator contacts between the five- and 10-hermit crab treatments compared to the zero-hermit crab treatment (GLM, *F*_2,68_ = 1.74, *p* = 0.182, $\eta_{p}^{2}$ = 0.04; figure 7c). We did see an effect of season, with all contacts in the summer and none in winter/spring (*F*_1,68_ = 5.07, *p* = 0.028, $\eta_{p}^{2}$ = 0.06). There was no interaction between treatment and season (*F*_2,68_ = 1.25, *p* = 0.292, $\eta_{p}^{2}$ = 0.03). There was also no effect of water temperature (*F*_1,68_ = 0.09, *p* = 0.346, $\eta_{p}^{2}$ = 0.01).

## 4. Discussion

In our first experiment, we saw that more primary scavengers were attracted to the accessible mussel carcass than the inaccessible carcass or the no-carcass control. We also found that more secondary scavengers were seen in the accessible carcass treatment than the inaccessible or control treatments. Secondary scavengers were those that we did not see feeding directly from the carcass, but which, we speculate based on earlier work [21], were drawn to particles detached through the feeding actions of the hermit crabs. We did not see more secondary scavengers in the inaccessible carcass treatment compared to when no carcass was present at all, implying that primary scavenger activity, feeding on and releasing material from the carcass, rather than the carcass itself, is what drew the secondary scavengers to the area. Here, we also saw an interaction between season and treatment, recording more secondary scavengers at the accessible carcass during the spring/summer season than in winter. Two-spot gobies were common visitors in both seasons, but in the spring/summer we saw more mysids, and most abundantly, juvenile whiting, which were completely absent in the winter month trials. Seasonal effects are to be expected, though we caution against inferring too much from a study that only took place over 1 year. We speculate that the juvenile whiting use the rockpools as a refuge from predators [27], as a foraging area for seasonally occurring microorganisms around the shoreline, or to exploit the warmer temperatures of the rockpools during the spring/summer months compared to the open sea, a form of behavioural thermoregulation [28], perhaps facilitating faster development and growth [29].

In a similar experiment, Davenport *et al.* [10] described clouds of particles released by scavenging crabs feeding on small fish baits and similarly reasoned that these might also have attracted further foragers. Other cues may have been important too. Acoustic cues can carry over long distances underwater, and aggregations of scavengers in aquatic environments are known to produce sounds as they break up the carcass and compete with one another. Auster *et al.* [15] monitored scavenging of an experimentally sunken billfish carcass, reporting acoustic cues produced by small sharks as they tore the carcass with their teeth and beat their tails to twist and detach pieces of tissue. They suggested that these cues may attract other scavengers to the location of the carcass. In our study, tearing of the mussel tissues by the hermit crabs, along with ‘clicking’ sounds produced as their shells tapped together as they fed and fought over the food [30], may have provided acoustic social cues to other nearby scavengers.

In our second pair of experiments, we saw that while more hermit crabs visited the baited rig than the unbaited rig, the numbers of potential predator species recorded did not differ between the two treatments. This was surprising, since green shore crabs and shannies will feed directly on mussel baits, and we might have expected them to have been further attracted to these by the foraging activities of the hermit crabs. Even though overall predator visits did not differ between the treatments, we did record more predator attacks on scavengers at the baited compared to the unbaited treatment. This suggests that carcass scavenging might be costly for smaller scavengers, since it could increase the frequency with which they encounter predators. We emphasize though that the overall attack frequency by predators on scavengers was low in this study. Moreover, predators were far more likely to feed on the mussel bait than on the hermit crabs, suggesting they may act primarily as competitors rather than predators. It is possible that hermit crabs, protected by their gastropod shells, [31] are not as vulnerable to these predators as other, unarmoured small scavengers. This may explain why hermit crabs were the most commonly seen scavenger species, with more vulnerable species perhaps more likely to avoid carcasses in order to avoid predators, a similar phenomenon to the landscape of fear effects that influence scavenger distribution in terrestrial systems [16].

When we presented simulated aggregations of hermit crabs in stimulus chambers in the absence of carcass baits, we saw that green shore crabs and shannies were more likely to approach groups of five or 10 hermit crabs than they were to approach empty chambers. Attempted attacks on the confined hermit crabs were again rare. Predatory species may have perceived the aggregated hermit crabs as potential prey, but they might also have been responding to social cues from the hermit crabs. Aggregated hermit crabs are visually conspicuous, probably produce concentrated chemical cues and produce noise while physically interacting [30]. All of these cues might be associated with feeding interactions, and approaches by green shore crabs, shannies and other fishes might reflect local enhancement of a potential feeding site [32], rather than predation attempts, although if this were the case we might have expected to see more hermit crabs approach these aggregations too.

While we observed few predation attempts, it may be that at low tide and during daylight hours, when we conducted our experiments, predation pressure is simply less intense. Larger predatory fish are absent at low tide, and lobsters (*Homarus gammarus*), larger crustacean predators, though present, are mainly active at night [33]. The level of risk faced by hermit crabs and other scavengers might be significantly greater when the rockpools are inundated and a more diverse assemblage of predators, many larger and better equipped to handle hard-shelled prey, can access them. Future work might consider this by running similar experiments over the duration of the tidal cycle, in the sea proper and at different times of day.

In summary, we have shown that primary scavengers feeding on carcasses in rockpools are associated with an increase in the numbers of some fishes and mysids in the immediate location. The importance of scavenging in food web structure and trophic energy transfer has been previously underestimated [2,3,5]. We suggest that through their feeding action, primary scavengers made material from the carcass available to a wider range of species, including some not otherwise equipped to exploit carcasses directly. We have also shown that aggregations of scavengers are potentially at greater risk of predation. Although attacks were rare, they occurred more frequently at baited than unbaited sites, and predators approached clustered hermit crabs, even in the absence of carcasses. Future work will focus on how carcass size and persistence affect the rate at which larger predators visit carcasses and shape landscapes of fear, in turn affecting how smaller scavengers exploit or avoid carcass resources [16,34,35]. We suggest that rocky shore ecosystems represent a tractable system for more focussed experimental studies into the community ecology of scavenging.

## Supplementary Material



## Data Availability

The data generated by this project can be found in the supplementary material [36].
